# Clinical characteristics and genomic epidemiological survey of tuberculosis in Wuzhou, China, 2022

**DOI:** 10.1128/spectrum.02474-24

**Published:** 2025-04-10

**Authors:** Jianpeng Li, Manman Zhao, Weidao Huang, Xuanxuan Huang, Yongqiang Ou

**Affiliations:** 1 Wuzhou Third People’s Hospital, Wuzhou, Guangxi, China; 2 Medical School, Shenzhen University 481870, Shenzhen, Guangdong, China; ICON plc, London, United Kingdom

**Keywords:** tuberculosis, *Mycobacterium tuberculosis*, whole-genome sequencing, epidemiology, drug resistance

## Abstract

Tuberculosis (TB) is a serious respiratory disease posing significant public health threats, such as the variation in *Mycobacterium tuberculosis* (*M.tb*) lineages and their associated drug resistance across regions. In 2022, clinical data and culture-positive TB samples were collected from the Third People’s Hospital in Wuzhou, China. *M.tb* drug resistance and lineage were analyzed using whole-genome sequencing, while logistic regression was applied to identify factors influencing patient outcomes. Among 169 strains analyzed, an overall drug resistance rate of 23.1% was observed. Multidrug-resistant or rifampicin-resistant cases constituted 7.7% of the strains. Most strains belonged to lineage 2 (69.8%), followed by lineage 4 (27.8%). Poor treatment adherence, being aged 65 or older, and retreatment emerged as risk factors for unfavorable outcomes. This pioneering survey provides crucial insights into TB patient characteristics, drug resistance patterns, and lineage distribution in Wuzhou, laying a foundation for future targeted TB control strategies in the region.

**IMPORTANCE:**

In 2022, tuberculosis (TB) was the second leading cause of death from a single infectious agent worldwide, posing a serious threat to global health. The epidemiological characteristics of TB vary considerably from country to country, and even from region to region within a single country, due to differences in the economy, medical conditions, education, and other factors. Understanding the current status of TB epidemics in the region is important, with practical implications for local diagnosis, treatment, and control. This genomic epidemiological survey has provided a first insight into the characteristics of TB patients, drug resistance rates, prevalence lineage, and factors associated with unfavorable outcomes in Wuzhou, China.

## OBSERVATION

Tuberculosis (TB) is a respiratory infectious disease that poses a serious threat to public health worldwide (1). China is one of the high-TB-burden countries, with 748,000 new cases and 30,000 multidrug-resistant or rifampicin-resistant TB (MDR/RR TB) cases in 2022 (2). However, due to the vastness of China, the characteristics of TB epidemics differ from region to region in terms of economic development and population habits. It is important to understand the current status of TB epidemics in the region, which has practical implications for local diagnosis, treatment, and control.

The Guangxi Zhuang Autonomous Region (Guangxi for short), located in southern China, is the province with the fifth highest TB incidence in China, with an annual notified incidence rate of approximately 100 cases/100,000 (3). As expected, TB incidence also varies widely within Guangxi, with a high rate in the northern part (217.2/100,000) and a low rate in the southeastern part (31.2/100,000) (4). The city of Wuzhou, located in the easternmost part of Guangxi, is a moderately developed city with a TB incidence rate of 57.9/100,000. However, the current status of TB prevalence and drug resistance in Wuzhou remains unknown.

To investigate the current status of TB prevalence, we anonymously collected information and all culture-positive *Mycobacterium tuberculosis* (*M.tb)* strain whole-genome sequencing (WGS) data from 169 TB patients (one strain per TB patient) admitted to the only municipal TB-designated hospital in Wuzhou City in 2022 and conducted the first genomic epidemiological survey in this area. Because diagnostic tests were routinely used in clinical practice and all individual patient information was removed before analysis, the Ethics Committee of Wuzhou Third People’s Hospital waived the requirement of informed consent from individual patients.

Of the 169 TB patients, the majority were older than 45 years (68.6%, 116/169), 79.9% (135/168) were male, and 46.7% (79/168) lived in rural areas (Table 1). In addition to TB, 19.5% (33/168) of patients had comorbidities with diabetes, 7.7% (13/168) had comorbidities with hyperlipidemia, and 5.4% (9/168) had concurrent HIV infection. Of the patients, 88.6% (148/167) were new cases and 11.4% (19/167) were recurrent cases. The favorable outcome (cured or treatment completed) rate was 79.3% (134/169), while the unfavorable outcome (treatment failure, death, or lost to follow-up) rate was 20.7% (35/169).

**TABLE 1:** Demographic and clinical characteristics of 169 TB patients^*c*^

Characteristics (*n* = 169)	*n*	%
Gender (*n* = 168)		
Male	135	79.9
Female	33	19.5
Age		
<25	10	5.9
25–44	43	25.4
45–64	58	34.3
≥65	58	34.3
Residence (*n* = 168)		
Urban	79	46.7
Rural	67	39.6
Outside the city	22	13.0
Complications		
Diabetes	33	19.5
Hyperlipemia (*n* = 168)	13	7.7
HIV positive (*n* = 168)	9	5.4
Malnutrition (*n* = 168)	47	28.0
Living habits		
Smoking (*n* = 157)	84	53.5
Drinking (*n* = 157)	53	33.8
Treatment		
Retreated (*n* = 167)	19	11.4
Poor adherence (*n* = 156)	96	61.5
Unfavorable outcome	35	20.7
Drug resistance profile		
DS	130	76.9
MDR/RR^*a*^	13	7.7
pre-XDR	4	2.4
Other	26	15.4
Lineages		
Lineage 1	3	1.8
Lineage 2	118	69.8
Lineage 4	47	27.8
Lineages 2 and 4^*b*^	1	0.6

^*a*^ The number of MDR/RR cases includes pre-XDR cases.

^*b*^ Co-infection with *M.tb* lineages 2 and 4, indicating mixed infection.

^*c*^ Abbreviations: pre-XDR, pre-extensively drug-resistant; DS, drug susceptible.

**Fig 1:**
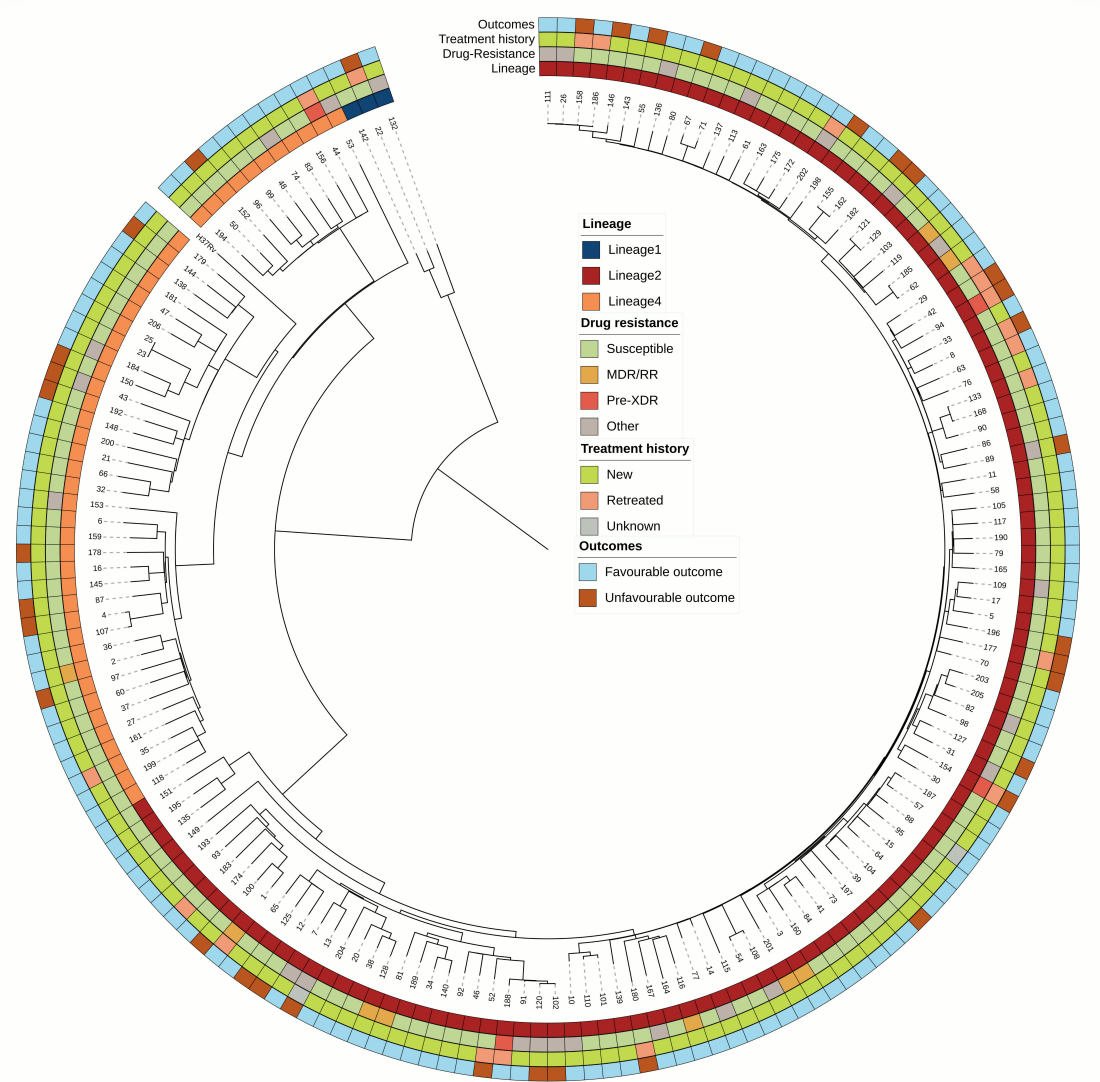
Phylogeny, lineage, treatment history, outcomes, and resistance profile of 168 *M*.*tb* strains. One mixed infection strain with lineages 2 and 4 excluded from phylogenetic analysis. Abbreviation: pre-XDR, pre-extensively drug-resistant.

Strain WGS data were used to analyze strain lineage, predicted drug resistance, and phylogenetic tree by the online platform SAM-TB (https://samtb.uni-medica.com) (5). The phylogenetic tree was constructed using the maximum likelihood method and annotated using iTOL (https://itol.embl.de/) (6). Of the 169 *M*.*tb* strains, 69.8% (118/169) belonged to lineage 2, 27.8% (47/169) to lineage 4, and only 1.8% (3/169) to lineage 1 (Fig. 1), with one strain being a mixed infection of lineages 2 and 4.

Drug resistance mutations were detected in 39 of the 169 strains, with an overall resistance rate of 23.1% (39/169). The resistance rates, from highest to lowest, were as follows: isoniazid (INH), 11.8% (20/169); streptomycin (STM), 8.9% (15/169); rifampicin (RIF), 7.7% (13/169); ethambutol (EMB), 6.5% (11/169); pyrazinamide (PZA), 4.1% (7/169); fluoroquinolones (FQs) (moxifloxacin [MFX] and levofloxacin [LFX]), 4.1% (7/169); ethionamide (ETO), 3.0% (5/169); aminoglycosides (AGs) (amikacin, kanamycin (KAN), and capreomycin), 1.2% (2/169); and p-aminosalicylic acid (PAS), 1.2% (2/169). No cycloserine, linezolid, clofazimine, bedaquiline, or delamanid resistance-associated mutations were detected.

Compared with drug-susceptible TB, MDR/RR TB requires longer treatment and more expensive but less effective antibiotics (7). In this study, a total of 13 MDR/RR (resistance to RIF) strains were detected (7.7%, 13/169), of which 4 were pre-extensively drug-resistant TB (pre-XDR, resistance to both RIF and FQs [8]) strains (30.8%, 4/13). Among the new TB patients, eight MDR/RR TB cases were detected (5.4%, 8/148), but no pre-XDR TB cases. Among the recurrent TB patients, five MDR/RR TB cases were detected (26.3%, 5/19), of which four were pre-XDR TB cases (80.0%, 4/5). In Hunan Chest Hospital, these proportions were 6.2% and 50.0% among new and recurrent TB patients, respectively, in 2009–2010 (9); in Beijing 309 Hospital, between 1997 and 2009, up to 14.7% of new TB patients and 36.9% of recurrent TB patients were MDR/RR TB (10). Compared with those hospitals in China, Wuzhou Third People’s Hospital had a lower rate of MDR/RR TB in both new and recurrent TB patients.

LFX and MFX of FQs are important components of the MDR/RR TB regimens (11). During the study period, there were seven FQ-resistant strains; 57.1% (4/7) were MDR/RR, 28.6% (2/7) were FQ mono-resistant strains, and 14.3% (1/7) were both FQ and STM resistant. We further inquired about the use of FQ drugs in these FQ-resistant patients and found that all of them had used FQs to treat bacterial infections other than TB. As TB retreatment is a recognized risk factor for FQ resistance (12), as expected, of the four recurrent FQ-resistant patients, three (two MDR/RR TB and one non-MDR/RR TB) had a clear history of TB treatment with FQs. FQs are broad-spectrum antibiotics that are commonly used to treat other microbial infections as well as TB (13). When FQs are used to treat other microbial infections in TB patients, short-term monotherapy is likely to result in acquired FQ resistance in *M.tb*. Due to the high level of cross-resistance between FQ drugs (14), the risk of developing FQ resistance remains even when using FQ drugs other than LFX and MFX. Due to the limited number of FQ-resistant patients in this study, more evidence and long-term investigations are needed to draw reliable conclusions about FQ-resistant TB in Wuzhou.

Among the INH-resistant strains, the most common mutation was *katG* S315T (65.0%, 13/20), followed by the *fabG1* C-15T mutation (10.0%, 2/20). Two strains had INH-resistant double mutations. One strain had double mutations of *fabG1* C-15T and *katG* W191R, and one strain had both a deletion at nucleotide position 1583 and an insertion at 1609 in *katG* (Table 2). All 13 RIF-resistant strains had mutations in the *rpoB* gene, with the highest proportion being S450L (30.8%, 4/13), and two RIF-resistant strains had multiple mutations (15.4%, 2/13). A total of five resistant mutation types were identified in all seven FQ-resistant strains, with the D94 codon mutation accounting for the highest proportion at 57.4% (4/7).

**TABLE 2:** Distribution of drug-resistant mutations

Drug	Gene	Mutation^*b*^	*n*	%
INH (*n* = 20)	*katG*	S315T	13	65.0
		S315N	1	5.0
		A1583del^*a*^, T1609TAins^*a*^	1	5.0
	*fabG1*	C-15T^*a*^	2	10.0
	*inhA*	S94A	1	5.0
	*ahpC*	G-48A^*a*^	1	5.0
	*fabG1*, *katG*	C-15T^*a*^ (*fabG1*), W191R (*katG*)	1	5.0
RIF (*n* = 13)	*ropB*	S450L^*a*^	4	30.8
		L430P	2	15.4
		S450W^*a*^	1	7.7
		D435Y^*a*^	1	7.7
		H445D^*a*^	1	7.7
		F208L	1	7.7
		A125V	1	7.7
		L430P, S431G	1	7.7
		L430P, D435E, T676P	1	7.7
EMB (*n* = 11)	*embA*, *embB*	C-12T^*a*^, Q497R	1	9.1
		C-16T^*a*^, Q497R	1	9.1
	*embB*	M306V	3	27.3
		M306I	2	18.2
		Q497R	2	18.2
		G406D	1	9.1
		G406S	1	9.1
PZA (*n* = 7)	*pncA*	A28D	1	14.3
		M175V	1	14.3
		M1T	1	14.3
		Q10P	1	14.3
		T-11G^*a*^	1	14.3
		429delG^*a*^, 524–525insTC^*a*^	2	28.6
STM (*n* = 15)	*rpsL*	K43R	7	46.7
		K88R	2	13.3
	*rrs*	G888A^*a*^	3	20.0
		A514C^*a*^	1	6.7
		C799T^*a*^, G888A^*a*^	1	6.7
	*gid*	102delC^*a*^	1	6.7
AGs (*n* = 2)	*rrs*	A1401G^*a*^	2	100
FQs (*n* = 7)	*gyrA*	A90V	2	28.6
		D94N	1	14.3
		D94G	1	14.3
		D94H	1	14.3
		P102H	1	14.3
		A90V, D94N	1	14.3
ETO (*n* = 5)	*fabG1*	C-15T^*a*^	3	60.0
	*ethA*	G43S	1	20.0
	*inhA*	S94A	1	20.0
PAS (*n* = 2)	*thyA*	H75N	2	100

^*a*^ Nucleotide mutation and position.

^*b*^ Amino acid mutation and position.

Univariate logistic regression analysis showed that age, treatment history, and drug adherence were significantly associated with unfavorable outcomes (Table S1). To exclude confounding factors, multivariate logistic regression analyses were performed on these factors (Table 3). The results showed that these factors remain associated with unfavorable outcomes. The factor with the highest odds of unfavorable outcomes was poor medication adherence (adjusted odds ratio [AOR] 13.81, 95% confidence interval [CI] 1.76–108.54, *P* = 0.001). In addition, the odds of unfavorable outcomes were 6.99 times higher for patients over 65 years of age compared with those aged 25–44 years (95% CI 2.32–21.04, *P* = 0.013), and 5.27 times higher for recurrent TB patients compared with new TB patients (95% CI 1.31–21.23, *P* = 0.019). None of the other factors, such as resistance profile, lineage, and comorbidities, were significantly associated with unfavorable outcomes (Table S1).

**TABLE 3:** Univariate and multivariate logistic regression analyses between outcomes and significantly associated factors

Characteristics	Favorable outcomes		Unfavorable outcomes		Total	OR	(95% CI)	*P* value	AOR	(95% CI)	*P* value
Age (*n* = 169)											
<25	10	100%	0	0%	10	NA^*a*^					
25–44	39	90.7%	4	9.3%	43	Ref			Ref		
45–64	48	82.8%	10	17.2%	58	2.03	(0.59–6.98)	0.260			
≥65	37	63.8%	21	36.2%	58	5.53	(1.73–17.65)	0.004	6.99	(2.32–21.04)	0.001
Treatment history (*n* = 167)											
New	123	83.1%	10	16.9%	148	Ref			Ref		
Retreated	10	52.6%	9	47.4%	19	4.43	(1.63–12.01)	0.003	5.27	(1.31–21.23)	0.019
Drug adherence (*n* = 156)											
Good	59	98.3%	1	1.7%	60	Ref			Ref		
Poor	73	76.0%	23	24.0%	96	18.59	(2.24–141.72)	0.005	13.81	(1.76–108.54)	0.013

^*a*^ NA, not applicable.

This genomic epidemiological survey has provided a first insight into the characteristics of TB patients, drug resistance rates, prevalence lineage, and factors associated with unfavorable outcomes in Wuzhou, China. It would provide a basis for future regional TB control.

## Data Availability

WGS data of *M.tb* strains are stored at the National Genomics Data Center of China (https://ngdc.cncb.ac.cn/) (BioProject accession: PRJCA030722).

## SUPPLEMENTAL MATERIAL

Table S1 spectrum.02474-24-s0001.docx Univariate logistic regression analysis of characteristics with outcomes.

ASM does not own the copyrights to Supplemental Material that may be linked to, or accessed through, an article. The authors have granted ASM a non-exclusive, world-wide license to publish the Supplemental Material files. Please contact the corresponding author directly for reuse.
