# Supplementary material for: Clinical characteristics and genomic epidemiological survey of tuberculosis in Wuzhou, China, 2022
Source: Microbiol Spectr. 2025 Apr 10;13(5):e02474-24. doi: 10.1128/spectrum.02474-24 (PMC12054142; doi:10.1128/spectrum.02474-24)
Supplement: Table S1 — Univariate logistic regression analysis of characteristics with outcomes. [file spectrum.02474-24-s0001.docx]

**Supplementary Table 1 Univariate logistic regression analysis of characteristics with outcomes.**

| **Characteristics (n=169)** | **Favourable outcomes** | | **Unfavourable outcomes** | | **Total** | **OR** | **(95% CI)** | ***P***  **Value** |
| --- | --- | --- | --- | --- | --- | --- | --- | --- |
| **Gender** (n=168) |  |  |  |  |  |  |  |  |
| Male | 25 | 75.8% | 8 | 24.2% | 33 | ref |  |  |
| Female | 109 | 80.7% | 26 | 19.3% | 135 | 0.75 | (0.30-1.84) | 0.524 |
| **Age** |  |  |  |  |  |  |  |  |
| <25 | 10 | 100% | 0 | 0% | 10 | NA |  |  |
| 25–44 | 39 | 90.7% | 4 | 9.3% | 43 | ref |  |  |
| 45–64 | 48 | 82.8% | 10 | 17.2% | 58 | 2.03 | (0.59-6.98) | 0.260 |
| ≥65 | 37 | 63.8% | 21 | 36.2% | 58 | 5.53 | (1.73-17.65) | 0.004 |
| **Residence** (n=168) |  |  |  |  |  |  |  |  |
| Urban | 60 | 76.0% | 19 | 24.1% | 79 | ref |  |  |
| Rural | 54 | 80.6% | 13 | 19.4% | 67 | 0.76 | (0.34-1.68) | 0.499 |
| Outside city | 19 | 86.4% | 3 | 13.6% | 22 | 0.50 | (0.53-1.87) | 0.302 |
| **Diabetes** |  |  |  |  |  |  |  |  |
| No | 109 | 80.2% | 27 | 19.9% | 136 | ref |  |  |
| Yes | 25 | 75.8% | 8 | 24.2% | 33 | 1.29 | (0.52-3.18) | 0.577 |
| **Hyperlipemia** (n=168) |  |  |  |  |  |  |  |  |
| No | 125 | 80.7% | 30 | 19.4% | 155 | ref |  |  |
| Yes | 9 | 69.2% | 4 | 30.8% | 13 | 1.85 | (0.53-6.42) | 0.331 |
| **HIV** (n=168) |  |  |  |  |  |  |  |  |
| No | 126 | 80.7% | 33 | 20.8% | 159 | ref |  |  |
| Yes | 9 | 69.2% | 1 | 11.1% | 9 | 0.48 | (0.06-3.95) | 0.493 |
| **Malnutrition** (n=168) |  |  |  |  |  |  |  |  |
| No | 101 | 83.5% | 20 | 16.5% | 121 | ref |  |  |
| Yes | 33 | 70.2% | 14 | 29.8% | 47 | 2.14 | (0.97-4.71) | 0.058 |
| **Smoking** (n=157) |  |  |  |  |  |  |  |  |
| No | 60 | 82.2% | 13 | 17.8% | 73 | ref |  |  |
| Yes | 65 | 77.4% | 19 | 22.6% | 84 | 1.35 | (0.61-2.97) | 0.456 |
| **Drinking** (n=157) |  |  |  |  |  |  |  |  |
| No | 85 | 81.7% | 19 | 18.3% | 104 | ref |  |  |
| Yes | 40 | 75.5% | 13 | 24.5% | 53 | 1.45 | (0.65-3.23) | 0.359 |
| **Treatment history** (n=167) |  |  |  |  |  |  |  |  |
| New | 123 | 83.1% | 10 | 16.9% | 148 | ref |  |  |
| Retreated | 10 | 52.6% | 9 | 47.4% | 19 | 4.43 | (1.63-12.01) | 0.003 |
| **Drug adherence** (n=156) |  |  |  |  |  |  |  |  |
| Good | 59 | 98.3% | 1 | 1.7% | 60 | ref |  |  |
| Poor | 73 | 76.0% | 23 | 24.0% | 96 | 18.59 | (2.24-141.72) | 0.005 |
| **DR-profile** |  |  |  |  |  |  |  |  |
| All susceptible | 103 | 79.2% | 27 | 20.8% | 130 | ref |  |  |
| Any resistant | 31 | 79.5% | 8 | 20.5% | 39 | 0.98 | (0.41-2.39) | 0.972 |
| **Lineage** |  |  |  |  |  |  |  |  |
| Lineage 1 | 2 | 66.7% | 1 | 33.3% | 3 | 2.11 | (0.17-25.93) | 0.559 |
| Lineage 2 | 93 | 78.8% | 25 | 21.2% | 118 | 1.14 | (0.49-2.66) | 0.770 |
| Lineage 4 | 38 | 80.9% | 9 | 19.2% | 47 | ref |  |  |
| Lineage 2&4^*^ | 1 | 100% | 0 | 0% | 1 | NA |  |  |

^*^ Co-infection with *M.tb* lineage 2 and lineage 4, indicating mixed infection.

Abbreviations: HIV, Human immunodeficiency virus; OR, odd ratios; CI, confidence interval; DR, drug resistance; NA, not Applicable.
